# Characterization and Antioxidant Properties of Six Algerian Propolis Extracts: Ethyl Acetate Extracts Inhibit Myeloperoxidase Activity

**DOI:** 10.3390/ijms15022327

**Published:** 2014-02-07

**Authors:** Yasmina Mokhtaria Boufadi, Jalal Soubhye, Ali Riazi, Alexandre Rousseau, Michel Vanhaeverbeek, Jean Nève, Karim Zouaoui Boudjeltia, Pierre Van Antwerpen

**Affiliations:** 1 Laboratory of Beneficial Microorganisms, Functional Food and Health (LMBAFS), Faculty of Natural Sciences and Life, University of Abdelhamid Ibn Badis, Mostaganem 27000, Algeria; E-Mails: yasminaboufadi@yahoo.fr (Y.M.B.); Ardz22003@yahoo.fr (A.R.); 2 Laboratory of Pharmaceutical Chemistry, Faculty of Pharmacy, Universite Libre de Bruxelles, Brussels 1050, Belgium; E-Mails: jal.sub@hotmail.com (J.S.); jneve@ulb.ac.be (J.N.); 3 Laboratory of Experimental Medicine, CHU Charleroi, A. Vésale Hospital, Universite Libre de Bruxelles, Montigny-le-Tilleul 6110, Belgium; E-Mails: Alexandre.ROUSSEAU@chu-charleroi.be (A.R.); michel.vanhaeverbeek@ulb.ac.be (M.V.); karim.zouaoui@chu-charleroi.be (K.Z.B.); 4 Analytical Platform of the Faculty of Pharmacy, Universite Libre de Bruxelles, Brussels 1050, Belgium

**Keywords:** propolis, oxidative stress, free radical scavenging, myeloperoxidase inhibition, LDL oxidation inhibition

## Abstract

Because propolis contains many types of antioxidant compounds such as polyphenols and flavonoids, it can be useful in preventing oxidative damages. Ethyl acetate extracts of propolis from several Algerian regions show high activity by scavenging free radicals, preventing lipid peroxidation and inhibiting myeloperoxidase (MPO). By fractioning and assaying ethyl acetate extracts, it was observed that both polyphenols and flavonoids contribute to these activities. A correlation was observed between the polyphenol content and the MPO inhibition. However, it seems that kaempferol, a flavonoid, contributes mainly to the MPO inhibition. This molecule is in a high amount in the ethyl acetate extract and demonstrates the best efficiency towards the enzyme with an inhibiting concentration at 50% of 4 ± 2 μM.

## 1. Introduction

Oxidative stress is the consequence of the imbalance between the production of reactive oxygen/nitrogen species (ROS/RNS) including: superoxide anion radical, singlet oxygen, alkoxyl radical, peroxyl radical, nitric oxide radical, peroxynitrite, hydroxyl radical, hydrogen peroxide and hypochlorous acid, and the antioxidant mechanisms which eliminate ROS and RNS [1]. These ROS/RNS promote oxidation of biomolecules such as DNA, RNA, proteins and lipids [2–4]. Alterations reported in these important biomolecules are involved in many inflammatory conditions such as in atherosclerosis, Parkinson’s disease, heart failure, myocardial infarction, Alzheimer’s disease, schizophrenia, cancer and fragile X syndrome [1,5,6]. Due to the possible oxidative damages resulting from ROS/RNS overproduction, the human body has developed many mechanisms of defense against these species. These antioxidant lines of defenses consist of antioxidant molecules and enzymes. Some of these compounds are components of normal diet such as vitamin C, vitamin E, polyphenols, flavonoids and carotenoids. When the supply of antioxidants is suboptimal, the activity of the antioxidant system is significantly affected [6].

Myeloperoxidase (MPO, EC 1.11.2.2) is one of the body enzymes that produces ROS. As a matter of fact, it catalyzes the formation of hypochlorous acid (HOCl) starting from hydrogen peroxide (H_2_O_2_) and chloride ions. This enzyme plays important roles in host defense; it is packed into azurophilic granules of neutrophils with a relatively high concentration, up to 5% of the dry weight of the cell and is released during phagocytosis where hypochlorous acid destroys invading pathogens [7]. Moreover, MPO triggers neutrophils by interacting with integrin receptor CD11b/CD18b [8].

**Figure 1.**
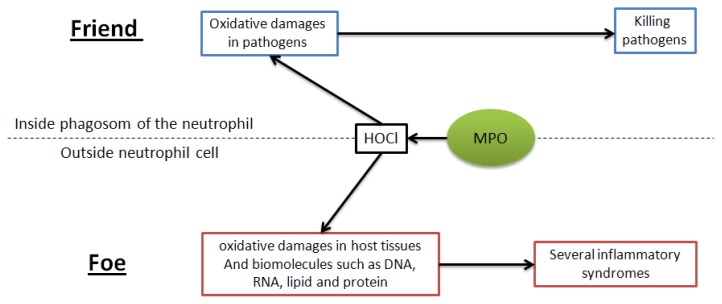
Myeloperoxidase (MPO) plays a critical role in the immune defense system by producing hypochlorous acid (HOCl) which contributes to killing pathogens. However, MPO can be poured out from neutrophils causing oxidative damage to the host tissues.

However, MPO has been located outside the neutrophils from where it can cause a wide spectrum of oxidative damage in host tissues by the oxidation of biomolecules (Figure 1) [7]. Indeed, during chronic inflammatory syndromes or a burst effect, neutrophils spontaneously degranulate or produce the so called, Neutrophil Extracellular Trap (NET) pouring out MPO in extracellular fluids [9].

The present study focuses on atherosclerosis which is a major problem for public health. The key role of MPO in atherosclerosis has been demonstrated as MPO can oxidize both low-density lipoprotein (LDL) and high-density lipoprotein (HDL), triggering endothelium inflammation and inhibiting the cholesterol efflux, respectively [10,11]. Moreover, MPO and its reaction products are present at all stages of the formation of atheroma plaque [12].

Because of the deleterious effects of HOCl, the inhibition of MPO has received much attention. Several compounds have been demonstrated to inhibit MPO: thioxantine [13], flavonoids [14,15], melatonin [16], aromatic hydroxamic acids [17], some anti-inflammatory drugs [18–20], tryptamines [21–23] and plant extracts, also the subject of many studies.

Propolis, or bee glue, is a vegetable putty which denotes a group of resinous substances [24], gummy and balsamic harvested by bees (*Apis mellifera*) foraging on bark [25] and buds mainly from various plants such as birch, poplars, oaks, willows, conifers and many others [26]. Propolis is a natural substance produced by bees from resins and gums; it is a mixture of salivary bee secretions and wax [26,27]. Recently, their curative properties were intensively studied through their antioxidant [24], anti-bacterial [28], anti-viral [29–31] and anti-cancer properties [32]. Many antioxidant compounds were found in propolis such as polyphenols, including phenolic acids and flavonoids (flavones, flavonols and flavanones) [33].

In this paper, we describe the antioxidant properties of propolis extract from different regions of Algeria. The content of polyphenols, flavonoids and ascorbic acid was assayed. The antioxidant effect of these compounds was determined by 2,2-diphenyl-1-picrylhydrazyl (DPPH) and lipid peroxidation scavenging. The inhibition of MPO as well as the inhibition of MPO-dependent LDL oxidation were also evaluated.

## 2. Results and Discussion

### 2.1. Assessment of Flavonoids, Polyphenols and Ascorbic Acid

**Figure 2.**
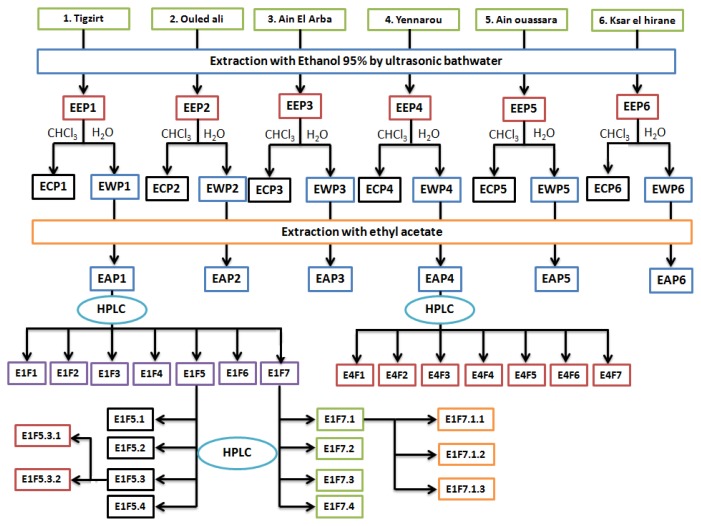
Extraction procedure and separation pathway for the propolis extract.

Table 1 summarizes the polyphenol compound concentrations in ethanol and acetyl acetate extracts (Figure 2). The extracts from Tigzirt (**1**) (EEP1 and EAP1) and Yennarou (**4**) (EEP4 and EAP4) showed the highest polyphenol concentrations, whereas the extracts taken from Ain ouassara (**5**) (EEP5 and EAP5) and Ksar el hirane (**6**) (EEP6 and EAP6) have the lowest concentrations. The concentrations of polyphenols in our samples are in the same order or higher than those reported in Greek regions by Kalogeropoulos *et al*. (80 mg gallic acid equivalents/g propolis) [34]. Compared to others studies, our results are in accordance with the polyphenol contents of propolis from different regions; Brazil: ~232 mg/g [35], China: 43–302 mg/g [36], India: 159–269 mg/g [37], Iran: 31–187 mg/g [38], Japan: 31–299 mg/g [32], South Korea: 161–213 mg/g [28], Portugal: 151–329 mg/g [39].

Flavonoid concentrations were also evaluated in each extract (Table 1). Similar results were observed. Extracts EEP1, EAP1, EEP4 and EAP4 showed the highest concentrations of flavonoids (69, 80, 56 and 72 mg/g of crude propolis, respectively) and ethyl acetate extracts appear to contain proportionally more flavonoids than the ethanol extracts. However, the flavonoid concentrations are comparable with data in the literature: the levels of flavonoids in propolis from different regions of South Korea range from 48 to 78 mg EQ/g of crude propolis [28]. A larger variability in flavonoid contents was shown in propolis collected in different regions of Iran 12–78 mg EQ/g [38]. In Argentina, Isla *et al*. demonstrated that the content of flavonoids varied between 14 and 62 mg/g [40]. According to Ahn *et al*. the flavonoid content of propolis from China is between 8 and 188 mg EQ/g of propolis [36]. Some authors, like Kumazawa *et al*. suggest the use of the flavonoid content as a criterion for differentiation between propolis [32].

Concerning ascorbic acid, Table 1 shows the concentrations in each of the extracts. Propolis of Tigzirt (**1**) and Yennarou (**4**) have the highest concentrations of ascorbic acid. According to these results, it was found that propolis of Tigzirt (**1**) and Yennarou (**4**) which are collected by Apis mellifica intermissa have the highest concentrations of antioxidant compounds including polyphenol, flavonoid and ascorbic acid. These compounds are found in lower concentrations in samples from Ain ouassara (**5**) and Ksar el hirane (**6**) which are collected by *Apis mellifica sahariensis*. The first two propolis were dark brown and taken from regions rich in plants such as crataegus, oak, lavender, eucalyptus and carob. Water is available in high percentage and the Mediterranean climate is found in these lands. However, the latter two samples have a yellow color and are collected from a poor location where plants and water are insufficiently provided. Thus, the types and the quantity of plants, the percentage of water, the climate and the type of bees might play an important role in the formulation of propolis.

### 2.2. Evaluation of Antioxidant Capacity

The antiradical scavenging properties of each extract were determined by the DPPH screening test and the results were expressed in equivalents of ascorbic acid (Table 2). EEP1 and EAP1 had the highest radical scavenging potency (*IC*_50_ = 19.4 and 16.3 μg/mL respectively, Tigzirt (**1**)) compared to ascorbic acid (*IC*_50_ = 3.1 μg/mL). The ethanolic and ethyl acetate extracts from the fourth crude propolis (EEP4 and EAP4, Yennarou (**4**)) showed also a high activity. However, extracts of propolis from Ouled ali (**2**), Ain Ouassara (**5**) and Ksar el Hirane (**6**) showed very high *IC*_50_ values. As a matter of fact, the ethanol extracts have higher *IC*_50_ values than those of ethyl acetate extracts. These results demonstrate that the extracts which contain a high amount of polyphenol, flavonoid, and ascorbic acid have a higher scavenging activity. Comparing our results of free radical scavenging with those in the literature, *IC*_50_ values in this study are in the range of those found in Portugal (6 μg/mL and 52 μg/mLfor propolis from Bornes and Fundado respectively [39]). The study of Brazilian propolis showed a scavenging activity of 40% or 57% at a concentration of 500 μg/mL [32,41]. These results also corroborate those recently observed by Benhanifa *et al*. (2013) and Piccinelli *et al*. (2013) with other Algerian propolis [42,43].

**Figure 3.**
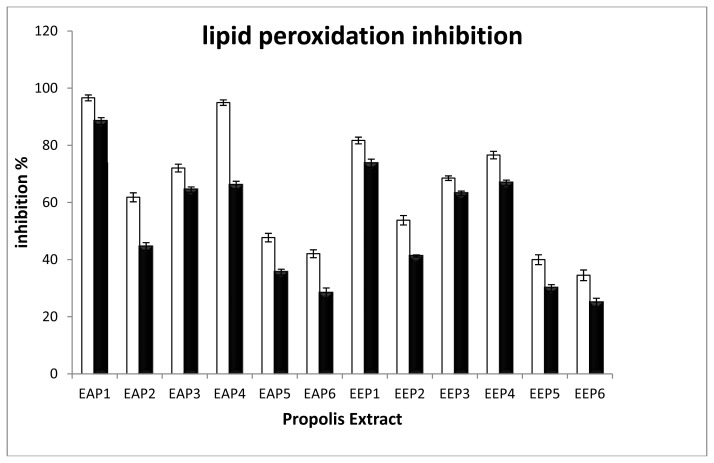
Percentage of lipid peroxidation inhibition. White bars indicate activities when the propolis extracts are combined within the liposomes (ST2), and the black bars illustrate lipid peroxidation inhibition when the propolis extract are added before H_2_O_2_ addition. The percentages of inhibition were calculated taking the control C1 and C2 as respectively 0% and 100% of inhibition.

The lipid peroxidation inhibition activities of each extract were determined on liposomes. Figure 3 summarizes the percentages of lipid peroxidation inhibition in the presence of 100 μg/mL of extracts. These results showed that liposomes are protected from lipid peroxidation by propolis extracts. Moreover, a higher efficiency of the propolis extracts in ST2 solutions were observed compared to ST1 solutions. The major difference is the presence of the propolis extract during the liposome formation in ST2 whereas the propolis extract was added after liposome formation and before the addition of H_2_O_2_ in ST1. In this context, the ST2 solutions are characterized by the presence of antioxidant molecules in the phospholipid bilayer of liposomes, and they can protect more efficiently the lipids from peroxidation. The ethyl acetate extracts had also protective effects and seem to be more efficient than the ethanol extracts. Like the free radical scavenging, the propolis extract from Tigzirt (**1**) and Yennarou (**4**) had the best activity. One hundred μg/mL of ethyl acetate and ethanolic extracts of propolis type 1 inhibited the peroxidation at 97% and 82%, respectively. Propolis extracts from Ain Ouassara (**5**) and Ksar el Hirane (**6**) have the lowest activities (ethyl acetate extracts inhibited lipid peroxidation with 48% and 42%, respectively).

### 2.3. Assessment of the Inhibition of MPO and LDL Oxidation Inhibition Occurring by MPO

**Figure 4.**
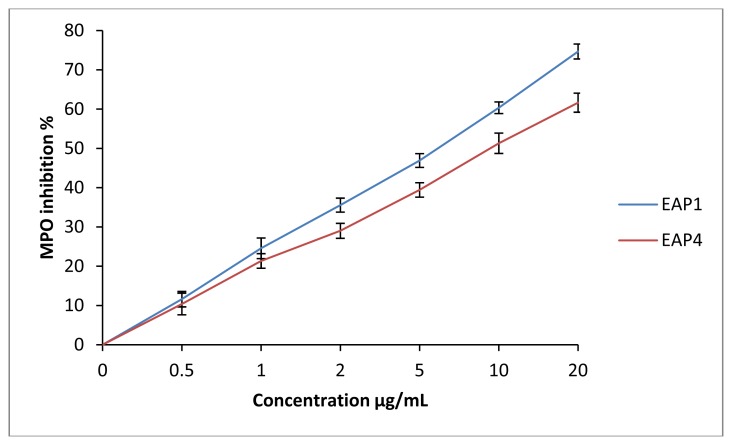
Percentage of inhibition of the MPO activity by ethylacetate extracts of propolis from Tigzirt (EAP1) and yennarou (EAP4).

Table 2 summaries the results of MPO inhibition of each ethyl acetate and ethanolic extract. Propolis extract from Tigzirt (**1**) and Yennarou (**4**) have the lowest IC_50_ values whereas propolis extracts from Ain Ouassara (**5**) and Ksar el Hirane (**6**) have the lowest activity (Figure 4). According to these results, there is a strong correlation between the polyphenol and flavonoid concentrations and the MPO inhibition. Indeed, polyphenols and flavonoids were reported to be efficient MPO inhibitors. Díaz-González *et al*. demonstrated that the polyphenol compounds of *Hamamelis virginiana* inhibit MPO at low concentrations [44]. Quercetin, which is a flavonoid, also demonstrated an efficient activity on the MPO (IC_50_ ≈ 5 μM).

One of the key roles of MPO in atherosclerosis is the oxidation of apolipoprotein B-100 of LDL that promotes endothelial inflammation and foam cells formation. Inhibition of MPO may prevent the oxidation of LDL and might reduce atherogenesis. Table 2 compares the percentages of MPO-dependent LDL oxidation inhibition with 20 μg/mL of extract and the IC_50_ values of MPO. These values are in the same range (~μg/mL) but the best extracts that inhibit LDL oxidation are EAP1 and EAP6. It is noteworthy that the percentage values for LDL oxidation are higher than MPO inhibition with the exception of EAP6. It has been suggested that MPO binds to LDL. This binding is thought to block the catalytic site of the enzyme which is located in a distal hydrophobic cavity with a narrow oval-shaped opening. This interference with the enzymatic inhibition may be the reason for the inhibitory effect change of the extracts between the MPO-mediated taurine chlorination and MPO-dependent LDL oxidation [45]. In addition, the large molecules of flavonoids and polyphenols may prevent them from easily entering the active site of MPO.

According to these results, the extracts EAP1 and EAP4 showed the highest activity on radical scavenging, lipid peroxidation inhibition and MPO inhibition. As a consequence, preparative LC was carried out on both extracts as described previously (Figure 2). Seven fractions were obtained for each extract and they were named accordingly (E1F1–7 and EAF1–7). Due to their high activity, the fractions E1F5 and E1F7 were separated under the same conditions.

Table 3 summarizes the polyphenol and flavonoid contents with the antiradical and MPO inhibition activities. The results demonstrate that all the fractions obtained from both EAP1 and EAP4 have high concentrations of polyphenols. However, among the fractions obtained from EAP1, the last three (E1F5–E1F7) have the highest amount of polyphenol while the first three fractions (E4F1–E4F3) of EAP4 featured the highest concentrations of polyphenols. These data suggest that the polyphenol content of EAP1 and EAP4 are different. Fractions obtained from EAP1 have high concentrations of flavonoids whereas only fraction E4F1 among the fractions obtained from EAP4 have a significant amount of flavonoids. The fractions of E1F5 and E1F7 were further fractioned by preparative LC and the concentrations of flavonoids in the resulting fractions decreased.

Fractions E1F5 to E1F7 obtained from EAP1, and E4F1 and E4F2 obtained from EAP4 can scavenge free radicals and inhibit MPO activity at low concentrations. Both E1F5 and E1F7 were further separated by preparative LC as they showed the best antioxidant activity with both systems. Table 3 showed that these subsequent fractions are also active. However, no correlation can be drawn between the flavonoid or polyphenol concentrations and their antioxidant activities. Indeed, fractions E4F3 to E4F7 are characterized by the absence of flavonoids and are less effective on MPO. As a matter of fact, flavonoids are also absent in fractions E1F5.1 and E1F5.2 but these fractions are characterized by an efficient inhibition of MPO. These results illustrates that both flavonoids and polyphenols are potentially able to inhibit MPO.

By considering the polyphenol and flavonoid contents of the different extracts and fractions, a correlation was observed between the polyphenol content and the inhibition of MPO activity whereas flavonoid content was not correlated to this inhibiting activity (See Figure S1). This observation could potentially explain the role of polyphenols and flavonoids on MPO inhibition. Indeed, the polyphenol concentrations in extracts and fractions are highly variable between samples and contribute to various inhibitions by scavenging effect. Conversely, the lack of correlation between flavonoid contents and MPO inhibition could illustrate that some flavonoids specifically inhibit MPO and others not.

The fractions, which have the highest activity and contain both flavonoids and polyphenols underwent LC/MS analysis to illustrate the compounds present in the active fractions of the propolis extracts. The compounds obtained from these fractions are listed in Table S1. All fractions were found to have polyphenol and flavonoid compounds except E1F7.1.2 where only polyphenols were detected. In addition to oleic acid, cinnamic acid and/or its derivatives can be found in most fractions. Tyrosol was found to be the principal polyphenol in our types of propolis fractions, while many types of flavonoids were found in the fractions such as chrysine, galangin, pinocembrin, quercetin, genistein and tectochrysin. All of these compounds were also found in the ethanolic extracts of propolis collected from Greece and Cyprus [34] (Table S1).

**Figure 5.**
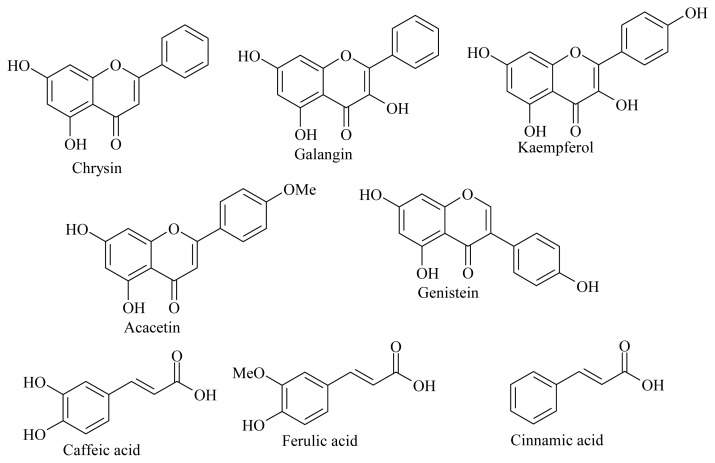
Structures of the tested compounds on MPO.

The compounds found in the most active fractions were tested for MPO activity (Figure 5). The *IC*_50_ values of caffeic acid, ferulic acid, chrysin, cinnamic acid, galangin, genistein, totarol, kaempferol, and acacetin were measured by the taurine chloramine assay (Table 4). The flavonoid derivative kaempferol showed the highest activity among all the tested compounds (*IC*_50_ of 4.1 ± 1.7 μM). Flavonoids such as chrysin and acacetin were less active. Among the polyphenols only ferulic acid showed a high activity on MPO. Meanwhile, cinnamic acid showed a good activity. It is worth mentioning that our results fit with the observations of Shiba *et al*. [14]. Indeed, the structure activity relationship obtained by Shiba *et al*. for the inhibition of MPO by flavonoids indicated that hydroxyl groups at the 3, 5, and 4′-positions and C2–C3 double bond are required for the inhibitory effect [14]. This structure-activity relationship can explain the *IC*_50_ values obtained in our work (Table 4) where it was also shown that flavonoid derivatives that have an OH group at position 3 and 4′ have a high activity on MPO. The flavonoid which has the phenyl group on position 3 instead of 2 showed a low activity. To sum up, these results suggest that the anti MPO activity of propolis extracts is due to several compounds derived from flavonoid, polyphenol and other structures.

## 3. Experimental Section

The solvents CH_2_Cl_2_, MeOH, EtOH, Butanol, ethyl acetate and CHCl_3_ were obtained from Sigma-Aldrich (St Louis, MO, USA). The reagents Folin-Ciocalteu, dichlorophenol-indophenol (DIP), 2,2-diphenyl-1-picrylhydrazyl (DPPH), phospholipids, thiobarbituric acid (TBA), tetramethoxypropane (TMP), taurine, 5-thio-2-nitrobenzoic acid (TNB), H_2_O_2_, NaOCl, and quercetin were also purchased from Sigma-Aldrich (St Louis, MO, USA). The following reagents were bought from Merck: NaHCO_3_, Na_2_CO_3_, AlCl_3_, ascorbic acid and phosphotungstic acid. HCl and H_2_SO_4_ were from VWR (Leuven, Belgium).

### 3.1. Collection, Extraction and Separation

The different types of propolis collected during the spring 2011 after the honey flow were supplied by beekeepers from different regions of Algeria as following: Tigzirt (**1**), Ouled ali (**2**), Ain El Arba (**3**), Yennarou (**4**), Ain ouassara (**5**), Ksar el hirane (**6**). The first four collections were produced by bees from the race of *Apis mellifica intermissa*, and the last two collections were produced by bees from the race of *Apis mellifica sahariensis*. The collection was performed by the method of grids and propolis was stored at −18 °C until analysis.

Each crude propolis (10 g) was divided into small pieces, and then crushed and extracted three times with ethanol 95% (100 mL) in an ultrasonic water bath for 1.5 h. The suspension was then filtered through a Whatman No. 1 paper, the solvent was subsequently evaporated to dryness under reduced pressure at 60 °C. The remaining solids represent the ethanolic dry extracts of propolis (EEP1–6). Part of the ethanolic dry extracts (EEPs) were subsequently suspended in 200 mL of water and extracted with 200 mL of chloroform. The organic layer was discarded and the aqueous phases were then extracted with 200 mL of ethyl acetate (EtOAc) three times. The organic phases of EtOAc were collected and evaporated to obtain EAP1–6 dry extracts. The assays on the different dry extracts were performed after dissolution in milliQ water [46].

The separation of EAP1 and EAP4 fractions were performed on preparative chromatographic system (LC, Waters, Milford, MA, USA) using Symmetry C18 preparative column (7 μm, 19 × 150 mm) and formic acid (2% in water)/methanol as mobile phase. The separations were performed in 90 min for each extract with a gradient according to the following time schedule (methanol %, time): 5%, 0 min.; 95%, 70 min.; and 30%, 90 min. The flow rate used was 5 mL/min. Seven fractions (E1F1–7 and E4F1–7) were obtained from both extracts (EEP1 and 4). The same LC procedure was used in order to separate the fractions, obtained by the previous process, which showed high activity on MPO. The mass spectra were obtained with a QTOF6520 (Agilent, Palo Alto, CA, USA) by using a column Zorbax Eclipse XDB C18 rapid resolution HT 4.6 × 50 mm, 1.8 μm, in ESI-positive mode, with a flow rate of 0.4 mL/min, with mobile phase ammonium acetate 10 mM (solution A): CH_3_OH (solution B) in gradient mode as following (B%, time): (10%, 0 min), (95%, 10 min), (10%, 15 min), (VCAP 3500 eV; Source T°, 350 °C; fragmentor, 110 V; skimmer, 65 V) (Figure 2).

### 3.2. Assay of Polyphenols, Flavonoids and Ascorbic Acid

The total polyphenols were determined by a colorimetric method using Folin-Ciocalteu reagent [47]. Five hundred microliters of the sample was placed in a 10 mL volume tube and diluted to 5.0 mL with distilled water. Then 0.5 mL of Folin-Ciocalteu reagent was added to the diluted sample. After 3 min, 0.5 mL Na_2_CO_3_ (10%) was added to the resulting mixture which was kept for 1 h in dark at room temperature. The absorbance was measured at 760 nm against a blank solution which consists of 1 mL of methanol.

A standard curve was drawn by using the same reaction conditions with gallic acid at different concentrations (0.0078, 0.0156, 0.0312, 0.0625, 0.125, 0.25, 0.5, 1 and 2 mg/mL). The total phenolic concentration was expressed as the mean ± SD in mg of gallic acid equivalent per g of crude propolis on three independent experiments.

Flavonoids in propolis extracts were determined by the method described by Woisky and Salatino [48]. One mL of aluminum chloride AlCl_3_ (2%) was added to 1.0 mL of sample, then the reaction was kept 30 min in the dark at room temperature. The absorbance was measured at 430 nm. Quercetin was used as standard and flavonoid content was expressed as mean ± SD mg of quercetin per g of crude propolis. Three independent experiments were done for each sample.

The assay of ascorbic acid was carried out by a colorimetric method [49,50]. Five hundred micrograms of crude propolis was cut into small pieces and extracted with 10.0 mL of oxalic acid (1%), then the extract was centrifuged at 3000× *g* for 15 min at room temperature. The resulting solution was mixed with 9.0 mL of dichlorophenol-indophenol (DIP) (0.05 mM in water) and the absorbance was measured at 515 nm after 15 s. The calibration curve was performed using a freshly prepared solution of ascorbic acid with concentration range from 0 to 500 μg using the same procedure as the samples. The results obtained from the ascorbic acid are expressed in mg/g of crude propolis. At least three independent experiments were done for each sample. The mean ± SD values were obtained.

Several molecules were assayed by LC/MS analysis. Briefly, EAP1 extract was dried and 1 mg was dissolved in milliQ water and then diluted to 0.1 mg/mL. The extract was analyzed by a LC procedure on a RRHD Zorbax eclipse plus C18 (2.1 × 50 mm, 1.8 μm) with a flow rate of 0.4 mL/min and mobile phase of formic acid 0.2% (solution A): CH_3_OH (solution B) in gradient mode as following (B%, time): (10%, 0 min), (10%, 0.5 min.), (80%, 15 min), (80%, 25 min), (10%, 25.5 min.) and (10%, 30 min). The data were acquired by a high resolution mass spectrometry QTOF6520 (Agilent, Palo Alto, CA, USA) in ESI-positive mode, (VCAP 3500 eV; Source T°, 350 °C; fragmentor, 110 V; skimmer, 65 V, mass range (100–1100 *m*/*z*). The concentrations were calculated with a calibration curve from 0.1 to 20 μg/mL.

### 3.3. Evaluation of Antiradical Activity by 2,2-Diphenyl-1-picrylhydrazyl (DPPH)

Evaluation of antioxidant capacity of propolis extracts was performed by the colorimetric method described by Arnous *et al*. [51]. According to this method the antiradical power of the extract is determined. The chemical compound 2,2-diphenyl-1-picrylhydrazyl (DPPH) was one of the first free radicals used to study the structure-activity relationship of antioxidant phenolic compounds [52,53]. It has an unpaired electron on a nitrogen atom of the bridge. Due to this relocation, the radical molecules do not form dimers and remain in its monomeric form relatively stable at room temperature and characterized by a blue color.

In this procedure, all samples were diluted 1:10 with MeOH immediately before the analysis. An aliquot of 0.025 mL of diluted sample (final concentration 0, 5, 10, 20, and 50 μg/mL) was added to 0.975 mL DPPH^•^ solution (60 mM in MeOH), and mixed with vortex, then the absorbance was measured at 515 nm after 30 min. The absorbance readings were converted to rates of DPPH radical-scavenging at time by the equation: [% RSA = (Abscontrole − Abst)/Abscontrole × 100]. Ascorbic acid was used as standard. The results were expressed as means ± SD for at least three independent experiments.

### 3.4. Scavenging Lipid Peroxidation

The scavenging of lipid peroxidation was carried out on liposomes in which propolis extracts were added during or after liposome formation. Briefly, four types of samples were prepared, two controls (C1, C2) and two tubes for samples (ST1, ST2). Ten mg of phospholipids from egg yolk were suspended in 10 mL of CH_2_Cl_2_/CH_3_OH 50:50 and 100 μL of the samples (EAP1–6) were added to the solution in the tubes (ST2). The solvent was evaporated to obtain a phospholipid film which was suspended with 2.0 mL of PBS buffer (50 mM, pH 7.4), producing liposomes. One hundred and ninety μL of each solution (C1, C2, ST1 & ST2) were taken. To the tube ST1 10 μL of the aqueous solutions of EAP1–6 were added while 10 μL of milliQ water were added to tube C1, C2 and ST2. After addition of 30 μL of H_2_O_2_ (1 mM) to tube C1, ST1 and ST2 and 30 μL of H_2_O to tube C2, the samples were allowed to stand 3 h at room light.

Lipid peroxidation was assayed according to Yagi, (1976) [54]. One hundred μL of each liposome suspension was taken and 4 mL of 1/12 N H_2_SO_4_ was added. After stirring, 500 μL of phosphotungstic acid solution (10%) was added. The resulting solution was left 5 min in darkness. Centrifugation at 1600 rpm was done over 10 min. The supernatant was removed and the residue was suspended in 2 mL H_2_SO_4_, then 300 μL of phosphotungstic acid was added. Another centrifugation at 1600 rpm was carried out over 10 min. The supernatant was discarded and the resulting sediment was dissolved in 4 mL of distilled water and 1 mL of TBA reagent (335 mg of thiobarbituric acid in 50 mL water/50 mL of acetic acid 99%). The resulting mixture was heated in water bath at 95 °C for 60 min. The solution was cooled and extracted with 5.0 mL of butanol. The fluorescence of the butanolic phase was measured with an excitation wavelength at 515 nm, an emission wavelength at 553 nm, the slide was 10 nm with the cut off at 515 nm. The quantity of lipid peroxidation was expressed as MDA equivalent from a linear curve drawn with several concentrations of the tetramethoxypropane (TMP) standard [54].

### 3.5. Preparation of the Recombinant Enzyme and of LDL

The recombinant MPO was produced as previously described Moguilevsky *et al*. and each batch solution was characterized by its protein concentration (mg/mL), its activity (U/mL), and its specific activity (U/mg). The chlorination activity was determined according to Hewson and Hager, (1979) [55]. Human plasma served for the isolation of LDL by ultracentrifugation according to Havel *et al*. (1955) [56]. Before oxidation, the LDL fraction (1.019 < *d* < 1.067 g/mL) was desalted by two consecutive passages through PD10 gel-filtration columns (Amersham Biosciences, Hoevelaken, The Netherlands) using PBS buffer. The different steps were carried out in the dark, and the protein concentration was measured by the Lowry assay for both MPO and LDL [57].

### 3.6. Myeloperoxidase Inhibition Assay

The assay was based on the production of taurine chloramine by the MPO/H_2_O_2_/Cl^−^ system which can be determined by means of 5-thio-2-nitrobenzoic acid (TNB) in the presence of a selected inhibitor at defined concentration. The reaction mixture contained the following reagents in a final volume of 200 μL:pH 7.4 phosphate buffer (10 mM PO_4_^3−^/300 mM NaCl), taurine (15 mM), extraction solutions of propolis, and the fixed amount of the recombinant MPO (6 μL of MPO batch solution diluted 2.5 times, 40 nM). When necessary, the volume was adjusted with water. This mixture was incubated at 37 °C and the reaction initiated with 10.0 μL of H_2_O_2_ (100 μM). After 5 min, the reaction was stopped by the addition of 10 μL of catalase (8 U/μL). To determine the amount of taurine chloramines produced, 50 μL of 1.35 mM solution of TNB were added and the volume adjusted to 300 μL with water. Then, the absorbance of the solutions was measured at 412 nm with a microplate reader and the curve of the absorbance as a function of the inhibitor concentration was plotted. To remove the influence of HOCl scavenging carried out by the extracts, the same procedure of MPO inhibition assay was done but with addition of 6 μL of HOCl (60 μM) instead of the solution of MPO and H_2_O_2_. The difference in the absorbance between the two tests was calculated and the *IC*_50_ values were then determined using standard procedures taking into account the absence of hydrogen peroxide as 100% of inhibition and the absence of inhibitors as 0% of inhibition [21].

### 3.7. Inhibition of LDL Oxidation

LDL oxidation was carried out at 37 °C in a final volume of 500 μL. The reaction mixture contained the following reagents at the final concentrations indicated between brackets: pH 7.2, PBS buffer, MPO (1 μg/mL), LDL (1000 μg/mL), 2 μL 1 N HCl (4 mM), one of the extracts at a different concentration, and H_2_O_2_ (100 μM). The reaction was stopped after 5 min by cooling the tubes in ice. The assay was performed as described by Moguilevsky *et al*. in a NUNC maxisorp plate (VWR, Zaventem, Belgium): 200 ng/well of LDL was coated overnight at 4 °C in a sodium bicarbonate pH 9.8 buffer (100 μL) [57], Afterward, the plate was washed with TBS 80 buffer and then saturated during 1 h at 37 °C with the PBS buffer containing 1% BSA (150 μL/well). After washing the wells twice with the TBS 80 buffer, the monoclonal antibody Mab AG9 (200 ng/well) obtained according to a standard protocol and as previously described was added as a diluted solution in PBS buffer with 0.5% BSA and 0.1% of Polysorbate 20. After incubation for 1 h at 37 °C, the plate was washed 4 times with the TBS 80 buffer and a 3000 times diluted solution of IgG antimouse alkaline phosphatase (Promega, Leiden, The Netherlands) in the same buffer was added (100 μL/well). The wells were washed again 4 times and a revelation solution (150 μL/well) containing 5 mg of para-nitrophenyl phosphate in 5 mL of diethanolamine buffer was added for 30 min at room temperature. The reaction was stopped with 60 μL/well of 3 N NaOH solution. The measurement of the absorbance was performed at 405 nm with a background correction at 655 nm with a Bio-Rad photometer for a 96-well plate (Bio-Rad laboratories, Hercules, CA, USA). Results were expressed as the percentages of Mox-LDL at 20 μg/mL of EAP [45].

### 3.8. Statistics

SigmaStat^®^ software (SPSS, 3.0, SPSS, Inc., Chicago, IL, USA) was used for the analysis. Data are presented as mean ± SD and were evaluated by one-way ANOVA, with Dunnett’s *post-hoc* test. When appropriate, an ANOVA on Rank with Dunn’s *post-hoc* test was used.

## 4. Conclusions

Many compounds were found in propolis extracts including flavonoids, polyphenols and other compounds such as cinnamic acid. The amounts of these compounds are affected by the types and number of plants, the availability of water and the climate. Propolis extracts, containing the higher amounts of polyphenols, flavonoids, and ascorbic acid, have the best antioxidant properties with the models proposed in this manuscript. Moreover, the ethyl acetate extraction seems to be more effective to obtain active extracts. Combination of all types of compounds appears to be important to raise an antioxidant activity that prevents the damage produced by oxidative stress. Some of these compounds work as radical scavenging and lipid antioxidants and others play an important role in MPO or LDL oxidation inhibition. Actually, the exact role in prevention of the oxidative damage of each compound has not yet been determined. However and because of its antioxidant potency, ethyl acetate propolis extract could be used as a natural medicinal agent when delivered in a suitable pharmaceutical form.

## Supplementary Information



## Figures and Tables

**Table 1.** Concentrations of polyphenols (Equivalent Acid Gallic, EAG), flavonoids (Equivalent Quercetin, EQ), and ascorbic acid in the ethanolic and ethyl acetate extracts of different types of propolis.

Propolis Sites	Sample	Total polyphenols (mg EAG/g)		Flavonoids (mg EQ/g)		Ascorbic acid (×10^−3^ mg/g)

		EEP	EAP	EEP	EAP	
**Tigzirt** §	1	279 ± 1	293.0 ± 0.5	69 ± 1	80 ± 1	0.94 ± 0.01
**Ouled ali**	2	125 ± 1	152 ± 1	36 ± 1	40.7 ± 0.9	0.45 ± 0.02
**Ain El Arba**	3	155 ± 1	194 ± 1	45 ± 1	58 ± 1	0.54 ± 0.02
**Yennarou** §	4	205 ± 2	223 ± 1	56 ± 1	71.6 ± 0.8	0.79 ± 0.02
**Ain ouassara**	5	55 ± 1	70 ± 1	24 ± 1	32 ± 1	0.39 ± 0.01
**Ksar el hirane**	6	75 ± 1	91 ± 1	10 ± 1	14.4 ± 0.9	0.29 ± 0.01

§ The content of polyphenols, flavonoids and ascorbic acid are significantly higher for Tigzirt and Yennarou extract compared to the other extracts (*p* < 0.05, Bonferroni’s test).

**Table 2.** Antiradical activity and Myeloperoxidase (MPO) inhibition activity of ethanol and ethyl acetate extracts, *IC*_50_ value of ascorbic acid as antiradical scavenging is 3.1 ± 0.2 μg/mL.

Propolis Site	Sample	Anti-radical test *IC*_50_ (μg/mL)		MPO inhibition *IC*_50_ (μg/mL)		% Mox-LDL with 20 μg/mL of EAP

		EEP	EAP	EEP	EAP	
**Tigzirt**	1	19.4 ± 0.2	16.3 ± 0.3	21 ± 1	6.9 ± 0.2 §	80 ± 4 †
**Ouled ali**	2	>50	>50	46 ± 1	40 ± 1	92 ± 3
**Ain El Arba**	3	34 ± 1	27.4 ± 0.4	21.0 ± 0.8	49 ± 2	92 ± 2
**Yennarou**	4	24.7 ± 0.3	19.6 ± 0.4	10.3 ± 0.3	12.8 ± 0.7 §	99 ± 4
**Ain ouassara**	5	>50	>50	41.6 ± 0.3	53.2 ± 0.8	89 ± 2
**Ksar el hirane**	6	>50	>50	48.7 ± 0.7	49 ± 1	77 ± 2 †

§ The *IC*_50_ are significantly different from the other extracts (*p* < 0.05, Bonferroni’s test);

† The % are statistically different from the other extracts (*p* < 0.05, Dunnet’s *Post-Hoc* test).

**Table 3.** Concentrations of flavonoids and polyphenols, antiradical scavenging activity and MPO inhibition activity of propolis fractions obtained from propolis type 1 and 4.

Fraction	Total polyphenols (mg EAG/g)	Flavonoïds (mg EQ/g)	Antiradical *IC*_50_ μg/mL	MPO inhibition *IC*_50_ μg/mL
E1F1	72 ± 1	39 ± 8	>50	24.3 ± 0.2
E1F2	88 ± 2	4.0 ± 0.5	>50	43 ± 2
E1F3	107 ± 2	48.2 ± 0.54	>50	11.7 ± 0.1
E1F4	223 ± 2	47 ± 5	>50	12.5 ± 0.3
E1F5	313 ± 2	31 ± 3	16 ± 1	1.6 ± 0.1
E1F6	230 ± 1	8.0 ± 0.3	24 ± 1	4.7 ± 0.2
E1F7	337 ± 1	51 ± 6	13 ± 1	1.28 ± 0.06
E4F1	210 ± 1	17 ± 2	20 ± 1	2.49 ± 0.06
E4F2	180 ± 2	0	35 ± 2	11.5 ± 0.6
E4F3	64 ± 2	0	>50	26.1 ± 0.5
E4F4	0	0	>50	49.0 ± 0.1
E4F5	0	0	>50	48 ± 1
E4F6	0	0	>50	43 ± 2
E4F7	10 ± 1	0	>50	38.7 ± 0.3
E1F5.1	314 ± 2	0	9 ± 1	2.6 ± 0.06
E1F5.2	162.9 ± 0.9	0	25 ± 2	10.6 ± 0.1
E1E5.3	213 ± 1	5.0 ± 0.7	13 ± 2	16.7 ± 0.6
E1F5.4	259 ± 1	5.0 ± 0.7	8.3 ± 0.3	3.7 ± 0.1
E1F7.1	206 ± 1	5.1 ± 0.2	17.0 ± 0.2	3.3 ± 0.06
E1F7.2	252 ± 2	0.8 ± 0.4	13 ± 1	3.97 ± 0.06
E1F7.3	155 ± 1	5.0 ± 1	26 ± 1	5.0 ± 0.1
E1F7.4	280 ± 2	22 ± 2	10.0 ± 0.1	2.62 ± 0.07
E1F5.3.1	163 ± 1	0	>50	20.6 ± 0.8
E1F5.3.2	189 ± 1	0	>50	13.0 ± 0.6
E1F7.1.1	249 ± 1	15 ± 1	21 ± 1	18 ± 2
E1F7.1.2	239 ± 1	0.5 ± 0.2	12 ± 1	6.6 ± 0.1
E1F7.1.3	271 ± 2	31 ± 1	10.0 ± 0.7	4.06 ± 0.03

**Table 4.** *IC*_50_ values of the pure compounds found in the most active fractions for MPO compared to their concentration in the ethyl acetate extract 1 (Tigzirt, 1.0 mg of extract/mL of water) and their concentrations extrapolated at the *IC*_50_ concentration of the ethyl acetate extract 1 (Tigzirt, 21 μg/mL).

Family	Compound	*IC*_50_ (μM) ± SD	Conc (μM)	Conc *IC*_50_ (μM)
**Flavonoids**	Chrysin $	8 ± 2	5.2 ± 0.8	0.11 ± 0.02
	Galangin	>10		
	Genistein	>10		
	Kaempferol $	4 ± 2	284 ± 12	6.0 ± 0.3
	Acacetin	9 ± 2	7.5 ± 0.4	1.57 ± 0.08

**Polyphenols**	Caffeic acid	>10		
	Ferulic acid $	6 ± 3	67 ± 17	1.4 ± 0.4
	Totarol	>10		

**other**	Cinnamic acid	9 ± 4	1.3 ± 0.6	0.03± 0.01

$ *IC*_50_ are significantly different from the other molecules (*p* < 0.05, Bonferroni’s test).
