# Supplementary material for: Characterization and Antioxidant Properties of Six Algerian Propolis Extracts: Ethyl Acetate Extracts Inhibit Myeloperoxidase Activity
Source: Int J Mol Sci. 2014 Feb 7;15(2):2327–45. doi: 10.3390/ijms15022327 (PMC3958853; doi:10.3390/ijms15022327)
Supplement: Supplementary file 1 [file ijms-15-02327-s001.pdf]

Supplementary Information

Table S1. Compounds found in propolis fractions obtained by preparative-LC. The identification was performed by LC-HRMS comparing the masses with a homemade database.

Fractions	Compounds	Total Conc. mg/g	Rt	ms	*m/z*	Ion	ppm
**E1–F5**	**Phenol**						
	Tyrosol		7.536	138.0681	156.102	M NH_4^+	0.24
	Caffeic acid		7.891	180.0423	181.0495	M H^+	−0.24
	Prenyl caffeate		11.448	248.1053	249.1126	M H^+	1.95
	Ferulic acid methylester		11.956	208.0739	209.081	M H^+	2.05
	Isopentyl caffeate		15.14	250.1217	268.1555	M NH_4^+	4.75
	Totarol		17.946	286.2283	304.2621	M NH_4^+	−4.81
	**Flavonoids**	31.1					
	Acacetin		12.901	284.0686	285.0759	M H^+	0.43
	Quercetin		13.139	302.0436	303.0511	M H^+	3.1
	Genistein		13.19	270.0533	271.0605	M H^+	1.7
	Kaempferol		13.537	286.0483	287.0555	M H^+	2.22
	Nareng énine		13.63	272.0691	273.0763	M H^+	2.22
	methyl quercetin		13.772	316.0589	317.0661	M H^+	1.77
	Bis-methylated quercetin		14.97	330.0751	331.0823	M H^+	3.63
	Tectochrysin		15.151	268.0738	269.0812	M H^+	1.16
	Pinocembrin		16.016	256.0741	257.0812	M H^+	1.78
	Chrysine		16.124	254.0581	255.0654	M H^+	0.92
	**Others**						
	3-Hydroxy-4-methoxycinnamic acid		10.021	194.0574	195.0647	M H^+	−2.48
	Pinobanksin-3-acetate		13.183	314.0796	315.0869	M H^+	1.91
	Methoxy-pinobanksin		13.188	286.0847	287.092	M H^+	2.26
	9-Octadecanoic acid		21.2	282.2562	300.29	M NH_4^+	1.36
**E1–F5.1**	**Phenol**						
	Ferulic acid methylester		7.317	208.0738	226.1074	M NH_4^+	1.3
	Tyrosol		7.528	138.0684	156.1023	M NH_4^+	2.51
	Caffeic acid		7.881	180.0421	181.0494	M H^+	−1.09
	Coumaric acid		9.518	164.0473	165.0546	M H^+	0.15
	3-OH-4-methoxycinnamic acid		10.018	194.0578	195.0651	M H^+	−0.65
	Isopentyl caffeate		15.139	250.1224	268.1551	M NH^{4+}	7.75
	Prenyl caffeate		15.762	248.1058	249.113	M H^+	3.87

Table S1. *Cont.*

Fractions	Compounds	Total Conc. mg/g	Rt	ms	*m/z*	Ion	ppm
	**Flavonoids**	4.9					
	Acacetin		12.916	284.0688	285.0761	M H^+	1.05
	Quercetin		13.156	302.043	303.0503	M H^+	1.1
	Genistein		13.221	270.0533	271.0606	M H^+	1.76
	Kaempferol		13.553	286.0483	287.0555	M H^+	1.96
	Naringénine		13.642	272.0691	273.0764	M H^+	2.24
	Methyl quercetin		13.79	316.0586	317.0659	M H^+	1.08
	Bis-methylated quercetin		14.977	330.0746	331.0819	M H^+	2.01
	**Others**						
	Glucose		1.1	180.0627	203.0519	M NH_4^+	−3.84
	Coumaric acid methyl ester		5.654	178.0628	196.0966	M NH_4^+	−0.7
	Methoxy-pinobanksin		13.202	286.0845	287.0918	M H^+	1.4
	Pinobanksin-3-acetate		16.114	314.0797	315.0869	M H^+	2.36
	9-Octadecanoic acid		21.234	282.2567	300.2906	M NH_4^+	3.33
**E1-F5.4**	**phenol**						
	Tyrosol		7.528	138.0684	156.1022	M NH_4^+	1.91
	Isopentyl caffeate		15.152	250.1222	268.1553	M NH_4^+	6.7
	Prenyl caffeate		15.769	248.1053	249.1126	M H^+	2.11
	Caffeic acid		7.891	180.0421	181.0493	M H^+	−1.35
	**Flavonoids**	5.0					
	Pinocembrin		16.028	256.0744	257.0817	M H^+	3.27
	Pinobanksin-3-acetate		16.108	314.0791	315.0864	M H^+	0.23
	Bis-methylated quercitine		16.136	330.0736	331.0809	M H^+	−0.86
	Chrysin		16.509	254.0583	255.0654	M H^+	1.7
	genistein		16.905	270.0535	271.0607	M H^+	2.45
	**Others**						
	Pimaric acid		19.339	302.2257	303.2329	M H^+	3.49
	9-Octadecanoic acid		21.259	282.2569	300.2907	M NH_4^+	3.83
**E1-F7.1.1**	**phenol**						
	Tyrosol		7.514	138.0688	156.1026	M NH_4^+	4.79
	Férulic acid		10.554	194.058	195.0653	M H^+	0.6
	Resveratrol		11.367	228.0788	246.1126	M NH_4^+	−0.84
	**Flavonods**	15.4					
	Galangin		14.516	270.0534	271.0607	M H^+	−2.18
	**Others**						
	9-Octadecanoic acid		21.265	282.2563	300.2901	M NH_4^+	−1.18
**E1-F7.1.2**	**phenol**						
	Tyrosol		7.527	138.0683	156.1022	M NH_4^+	1.73
	Isopentyl caffeate		15.162	250.121	268.1548	M NH_4^+	1.91

Table S1. *Cont.*

Fractions	Compounds	Total Conc. mg/g	Rt	ms	*m/z*	Ion	ppm
	**Others**						
	9-Octadecanoic acid		19.243	282.2563	300.2901	M NH_4^+	1.73
**E1-F7.1.3**	**phenol**						
	Tyrosol		7.32	138.0687	156.1025	M NH_4^+	4.05
	Caffeic acid		7.891	180.0422	181.0495	M H^+	−0.29
	Isopentyl caffeate		15.171	250.1211	268.155	M NH_4^+	2.51
	Ferulic acid methylester		3.51	208.0742	209.0815	M H^+	11.95
	**Flavonods**	30.6					
	Chrysin		16.517	254.0583	255.0655	M H^+	1.38
	Pinobanksin-3-acetate		16.136	314.0798	315.087	M H^+	2.39
	**Others**						
	Cinnamic acid		0.727	148.0523	166.0861	M NH_4^+	−0.78
	Ferulic acid methylester		11.953	208.0742	209.0815	M H^+	3.51
	Octadecanoic acid		18.445	282.2563	300.2901	M NH_4^+	1.6
	Pimaric acid		19.329	302.2248	303.2321	M H^+	0.69
**E1-F7.2**	**Phenol**						
	Tyrosol		7.522	138.0687	156.1025	M NH_4^+	4.22
	Totarol		16.878	286.2299	304.2634	M NH_4^+	0.73
	Prenyl caffeate		15.982	248.1057	249.113	M H^+	3.8
	**Flavonoids**	0.8					
	Kaempferol		13.537	286.0489	287.0561	M H^+	4.05
	methyl quercetin		13.77	316.0582	317.0655	M H^+	−0.35
	Genistein		14.471	270.0534	271.0607	M H^+	2.39
	Bis-methylated quercétine		14.967	330.0748	331.082	M H^+	2.62
	Acacetin		17.14	284.0689	285.0762	M H^+	1.53
	Pinostrobin		18.581	270.09	271.0973	M H^+	3.12
	**Others**						
	Pinobanksin-3-acetate		16.105	314.0787	315.0859	M H^+	−1.05
	Chrysin		16.506	254.0584	255.0657	M H^+	1.8
	Pimaric acid		19.361	302.2259	303.2332	M H^+	4.28
	9-Octadecanoic acid		21.294	282.2565	300.2903	M NH_4^+	2.54
**E1-F7.4**	**Phenol**						
	Tyrosol		7.527	138.0685	156.1023	M NH_4^+	3
	Isopentyl caffeate		15.138	250.1214	268.1553	M NH_4^+	3.62
	Totarol		19.412	286.2283	304.2621	M NH_4^+	−4.88
	**Flavonoids**	22.3					
	Tectochrysin		19.03	268.0741	269.0813	M H^+	2.18
	**Others**						
	Pimaric acid		19.361	302.2253	303.2326	M H^+	2.46
	9-Octadecanoic acid		21.262	282.2563	300.2901	M NH_4^+	1.81

Figure S1. Regression analysis between the polyphenol content of extracts/fractions and the MPO inhibition activity. Flavonoid content is not correlated with MPO inhibition activity (*R* = −0.04, *p* = 0.82).

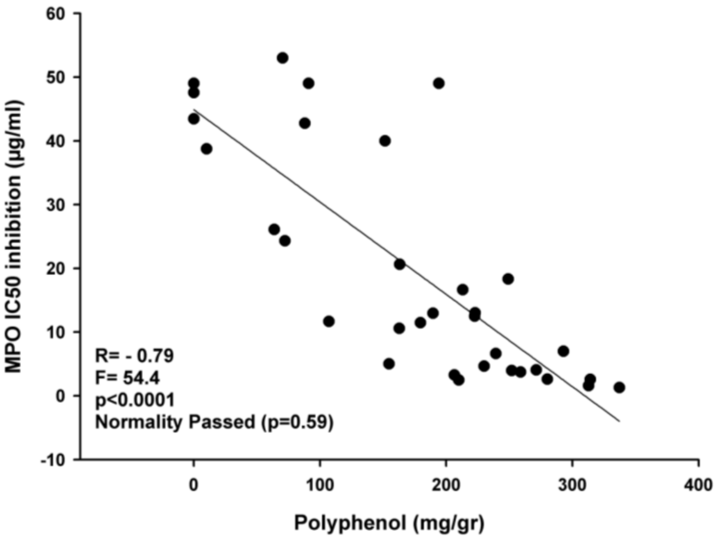

© 2014 by the authors; licensee MDPI, Basel, Switzerland. This article is an open access article distributed under the terms and conditions of the Creative Commons Attribution license (http://creativecommons.org/licenses/by/3.0/).
